# An innovative combination of cypropheptadine and prazosin for the treatment of alcohol use disorder: a double-blind, randomised, parallel-group, three-arm, multicentre, placebo-controlled phase 2 trial

**DOI:** 10.1192/j.eurpsy.2022.346

**Published:** 2022-09-01

**Authors:** H.-J. Aubin, A. Puech

**Affiliations:** Groupe Hospitalo-Universitaire AP-HP. Université Paris Saclay, Département De Psychiatrie Et D’addictologie, Villejuif, France

**Keywords:** Alcohol use disorder, Randomised Controled Trial, Treatment

## Abstract

**Introduction:**

Animal studies have shown that the simultaneous blockade of α1b-noradrenergic receptors and 5HT2A-serotonergic receptors strongly decreases alcohol intake. In addition, recent clinical studies have indicated that the selective α1b antagonist prazosin could be effective on alcohol use reduction in alcohol-dependent subjects.

**Objectives:**

Cocktail is a double-blind, randomised, parallel-group, three-arm, multicentre, placebo-controlled phase 2 proof-of-concept study aiming at demonstrating the superiority of a 12-week treatment with the KT110 combination of cyproheptadine (8 mg/day or 12 mg/day) and prazosin (5 mg/day or 10 mg/day) over placebo on the reduction of total alcohol consumption.

**Methods:**

The study two main inclusion criteria are a DSM5 diagnosis of severe alcohol use disorder and a WHO high-risk drinking risk level. The primary endpoint is the change from baseline (4 weeks preceding randomization) to the end of treatment (Weeks 9-12) in the mean quantity of alcohol consumed per day in the three groups. Daily alcohol consumption is determined using the Timeline Follow Back, automatically be filled in on the basis electronic patient reported outcomes platform. The 12-week treatment period is followed by a 4-week post-treatment follow-up.

**Results:**

One hundred and eighty patients are planned to be randomized 1:1:1 into the two treatment groups. Enrollment of patients started in November 2019, and will end in July 2021.

**Conclusions:**

In this communication, we will present the rationale for the development of the KT110 combination of cypropheptadine and prazosin for the treatment of alcohol use disorders, as well as the main features of the Coctkail study. ClinicalTrials.gov identifier: NCT04108104.

**Disclosure:**

Member of advisory boards, DSMB, or steering committees, speaker honoraria or consultancy for Bioprojet, D&A Pharma Ethypharm, and Kinnov Pharmaceuticals, Lundbeck, and Pfizer D&A Pharma, Ethypharm, Kinnov Pharmaceuticals, Lundbeck, and Pfizer.

